# Simultaneous Immobilization of Soil Cd(II) and As(V) by Fe-Modified Biochar

**DOI:** 10.3390/ijerph17030827

**Published:** 2020-01-28

**Authors:** Yi-min Wang, Shao-wei Wang, Cheng-qian Wang, Zhi-yuan Zhang, Jia-qi Zhang, Meng Meng, Ming Li, Minori Uchimiya, Xu-yin Yuan

**Affiliations:** 1 Key Laboratory of Integrated Regulation and Resource Development on Shallow Lakes, Ministry of Education, College of Environment, Hohai University, Nanjing 210098, China; wangym@hhu.edu.cn (Y.-m.W.); w1205788761@126.com (S.-w.W.); wsygsldxxn@163.com (C.-q.W.); zaysjzzyyj@163.com (Z.-y.Z.); zzjjqqhhu@163.com (J.-q.Z.); mmxxff1224@163.com (M.M.); 2 Huatian Nanjing Engineering & Technology Corporation, Nanjing 210019, China; 3 USDA-ARS Southern Regional Research Center, 1100 Robert E. Lee Boulevard, New Orleans, LA 70124, USA; sophie.uchimiya@usda.gov

**Keywords:** Cd-As contamination, biochar, metal forms, immobilization efficiency

## Abstract

Remediation of soil heavy metal by biochar has been extensively studied. However, few studies focused on the role of biochar on the co-immobilization of cadmium (Cd(II)) and arsenate (As(V)) and related soil nutrient availability. Remediation tests were conducted with three types of pristine and ferric trichloride (FeCl_3_) modified biochar (rice, wheat, and corn straw biochar) in Cd-As co-contaminated soil, with application rates of 1, 5, and 10% (w/w) and the incubation of 1, 7, 10, and 15 days. Using TCLP (Toxicity Characteristic Leaching Procedure) method, 10% of FeCl_3_ modified corn-straw derived biochar (FCB) had the highest immobilization efficiency of Cd(II) (63.21%) and As(V) (95.10%) after 10 days of the incubation. Iron-modified biochar immobilized higher fractions of water-soluble (F1) and surface-absorbed (F2) metal fractions than pristine biochar. For FCB amendment, Cd was mostly presented in the organic matter (OM) and sulfides associated (F4) and residual (F5) fractions (88.52%), as was found in the Fe-Al (oxides and hydroxides) (F3), F4, and F5 fractions (75.87%). FCB amendment increased soil pH values and available iron contents (*p* < 0.05), while no changes in soil available phosphorus content (*p* > 0.05). This study showed that FCB application reduces the environmental mobility of metals in Cd-As contaminated soil, while it also increases soil pH and available nutrient mobility, improving soil environmental quality and reducing remediation costs.

## 1. Introduction

As a non-essential element, cadmium Cd (II) is toxic to organisms at a trace concentration [1]. Excessive Cd(II) in soil is a risk not only to the food crops but higher organisms through the trophic transfer along the terrestrial food chain [1]. Accumulation of arsenite (As(III)) and arsenate (As(V)) oxyanions in soil and organisms are well described [2]. For example, in As contaminated mining districts (71–253 mg kg^−1^), As contents in the rice endosperm grain were up to 233–585 mg kg^−1^, which largely exceeded the level allowed in China (150 mg kg^−1^) in rice grain [3]. Due to the mining activities and pesticide usage, the co-contamination of Cd and As in soil poses serious threats to food safety [4]. Remediation of these co-contaminated soils has been an important issue for maintaining agriculture activities [5]. Due to the opposite chemical characteristics of Cd(II) and As(V), it is critical to find an efficient method for simultaneous remediating these co-contaminants. 

In comparison with other remediation materials, biochar derived from various solid organic wastes through pyrolysis under limited oxygen conditions has been extensively applied to treat heavy metal contaminated soil and water [6,7,8]. Due to its high specific surface area, negatively charged groups and porous structures, biochar is efficient in reducing the mobility of cationic heavy metal such as Cd(II) in soil through complexation, cation exchange, and precipitation [9,10,11]. The increased soil pH and mobility of dissolved silicon after biochar amendment not only reduced Cd(II) availability, but also decreased the Cd(II) uptake and translocation to plants [12,13,14]. Moreover, the soil-improving properties of biochar enabled its transfer into the conventional farming on a large scale in the future [15]. On the contrary, the adsorption capacity of biochar for anions like arsenate (As(V)) was significantly lower than those cationic toxics, mainly due to the negatively charged surface of biochar [16]. To facilitate the adsorption of As(V) onto biochar, iron oxides modification has been suggested [17,18]. Studies have proven that γ-Fe_2_O_3_ coated biochar significantly enhanced the sorption capacity of As(V) through the enhancement of electrostatic interactions [17,19]. Through incorporation of Fe into various biochar materials (e.g., empty fruit bunch, rice husk), amounts of absorbed As(V) increased by nearly 2 fold due to the formation of Fe-As complexes [20,21]. Therefore, Fe modification of biochar has been utilized for immobilization of As(V) contamination in soil. 

Unlike the behaviors of As(V) alone in soil, the co-existence of cationic toxics such as Cd^2+^, Zn^2+^ were reported to increase the adsorption of As(V) on magnetic-modified biochar, while it has no significant influence of Ag^+^ on the adsorption of As(V) is observed [22,23,24]. As a result, the role of Fe-modified biochar in affecting the mobility of heavy metals in multiple-contaminated soil is in confusion. Thus, we aimed to identify the feasibility of Fe-modified biochar on the remediation of Cd(II) and As(V). In this study, we characterized Fe-modified biochar materials, explored the Cd(II) and As(V) immobilization efficiency of biochar in soil, and evaluated changes of Cd(II) and As(V) fractions and soil properties after remediation. 

## 2. Materials and Methods 

### 2.1. Cd-As Contaminated Soil Preparation and Characterization

Soil samples from the 0–20 cm depths was collected from the rice-wheat rotation agriculture field in Xianning, Hubei province, China. The air-dried soil was sieved through a 2-mm mesh and homogenized before use. The soil contained sand (9.86%), silts (65.7%) and clay (24.4%). Cadmium nitrate (Cd(NO_3_)_2_) and sodium hydrogen arsenate (Na_2_HAsO_4_) were separately dissolved in the ultra-pure water and spiked to the homogenized soil to achieve the Cd-As co-contaminated soil. The concentrations of Cd (2.15 mg/kg) and As(V) (243 mg/kg) were chosen to represent a highly polluted area [25,26]. The spiked soil was aged in the greenhouse for three months. After that, the Cd-As aged soil samples were air dried, crushed and passed through 2 mm mesh sieve. 

Soil pH was measured at a solid-water ratio of 1:2.5 using a pH meter (Metler Toledo). Soil organic carbon (OM) content was determined by TOC Analyzer (Multi N/C 3000). Total nitrogen (TN) content was determined by the method of Kjeldahl [27]. Inorganic N was extracted with 1 M KCl from soil slurry [28]; ammonium and nitrite were analyzed by the phenol hypochlorite method [29], and the Griess-llosvay method [30], respectively. Soil available phosphorus (Olsen P) was extracted by 0.5 M NaHCO_3_, pH = 8.5 [31], soil total P was digested by a NaOH fusion method [32]. P content was measured using the molybdovando method described by Reuter and Robinson [33]. Soil total Cd and Fe content of soil samples were digested by HNO_3_-HCl-HF (4:1:1). DTPA (diethylene-triamine-pentaacetic acid) extraction method was used for analysis of soil available iron, denoted as DTPA-Fe [34,35]. All the digestion and extraction solutions were measured by ICP-OES (Prodigy, Leeman, USA). For As(V) in soil, microwave digestion (HNO_3_-H_2_O_2_) was employed to soil samples, and then measured by the atomic fluorophotometry AFS-2100 [36]. Soil characterization results are provided in the supporting information (Table S1).

### 2.2. Biochar Preparation, Modification and Characterization

Detailed pyrolysis processes were described in previous study [1]. Briefly, pristine biochar (BC) samples used in experiments were pyrolyzed from rice straw (RB), wheat straw (WB) and corn straw (CB) under a limited-oxygen condition using a patented slow-pyrolysis process (China Patent No. ZL200920232191.9) [37]. Straw materials were oven-dried for 12 h at 80 °C before moved to a reactor, which was heated by 5 °C min^–1^ to 400 °C under anaerobic conditions and then maintained for 4 h till no further smoke exhaust. Biochar was then ground and sieved through a 0.25-mm mesh before further application. To prepare the Fe-modified biochar (FBC), 10.0 g BC sample was mixed with 100 mL 0.3 M FeCl_3_ at pH 7.0 (0.5 mol/L of NaOH) for 2 h. The mixture was ultrasonic disperse for 2 h and then left at room temperature for 24 h. Then the mixture was dried at 80 °C for 48 h, after rinsed with deionized water for four times to remove free Fe^3+^ ion [24]. Then the dried Fe-modified BC powder was ground and sieved through a 0.25-mm mesh for future use. To characterize the properties of both BC and FBC, all samples were analyzed by SEM-EDS (S-4800, Hitachi, Japan) and the Fourier transform infrared spectroscopy (FTIR). 

### 2.3. Biochar Amendment on Cd-As Contaminated Soil

Incubation time and amendment dosage were first screened for the immobilization efficiency. 5% (w/w) BC and FBC were separately mixed fully with soil to obtain the biochar amended soils, and denoted as RB-, WB-, CB-, FRB-, FWB-, and FCB-soil. After that, the remediation tests were conducted with 10g of the amended soil or soil-only control treatments at a soil-to-water ratio of 1:1. This ratio had been used in the study of Sun et al. [38]. The mixtures were incubated for 1, 7, 10 and 15d at room temperature (25 ± 3 °C). Every three days added deionized water to maintain the soil-to-water ratio. Each treatment has three replications. The immobilization efficiency of Cd(II) or As(V) was evaluated using the TCLP (Toxicity Characteristic Leaching Procedure) methods following the Environmental Protection Industry Standard of China. In brief, 1.0 g incubated soil sample was mixed with 20 mL of acetic acid solution (pH = 2.50) at a solution-to-solid ratio of 20:1 [39]. Mixtures were rationally shaken for 10 h at 200 rpm. After centrifugation, the concentration of Cd in supernatants was measured by ICP-OES (Prodigy, Leeman, USA), As was measured by AFS-2100. After incubation, the Cd or As immobilization efficiency was calculated as follows [38]

Immobilization efficiency (%) = (1−M/M_0_)×100% (1)

where M is the quantity of extracted metal (Cd or As) in the supernatant and M_0_ is the quality of total As and Cd (mg) in the soil.

To investigate the role of amendment dosage on metal immobilization, 0%, 1%, 5% and 10% rate of BC or FBC were added to 10g Cd-As contaminated soil at a soil-to-water ratio of 1:1. The mixtures were incubated according to the optimal remediation time at room temperature as above mentioned. After that, the immobilization efficiency of metals was also calculated. 

### 2.4. Sequential Extraction of Cd and As from Soils 

A modified sequential extraction method was applied to partition the various species of both Cd and As in soil, which was adopted from Shiowatana et al. [40]. Metals were divided into five fractions as water-soluble metal (extracted by ultrapure water; F1: H_2_O-M); surface-adsorbed metal (extracted by 0.5M NaHCO_3_; F2: NaHCO_3_-M); Fe- and Al-associated metal (extracted by 0.1M NaOH; F3: NaOH-M); carbonate-bound metal (extracted by 1M HCl; F4: HCl-M) and residual metal (digested by HNO_3_-HF mixtures; F5: RS-M). The extracted supernatant is filtered through a 0.45-µm filter membrane. Cd content was analyzed by ICP-OES (ICAP7400, the lower detection limit is 0.05 mg/L) and As was measured by AFS-2100 (the lower detection limit is 0.2 μg/L). The quality control for Cd and As in soil was determined by measuring their content in the certified reference material (GBW 07405, the center of National Standard Reference of Material of China) [41]. This reference material was digested and measured concurrently as the soil samples did and obtained the recovery of Cd and As was 97.5 ± 2.7% and 101 ± 3.5%, respectively. 

### 2.5. Data Analysis

LSD (L) of one-way ANOVA was applied to assess the significant differences (*p* < 0.05) among groups using SPSS 21.0 (SPSS Inc., Chicago, IL, USA). Data are presented as mean ± standard deviation (*n* = 3). Charts and graphs were drawn by Sigmaplot 10.0 (Systat Software Inc., San Jose, CA, USA). 

## 3. Results and Discussion

### 3.1. Characterization of Biochar

**Figure 1:**
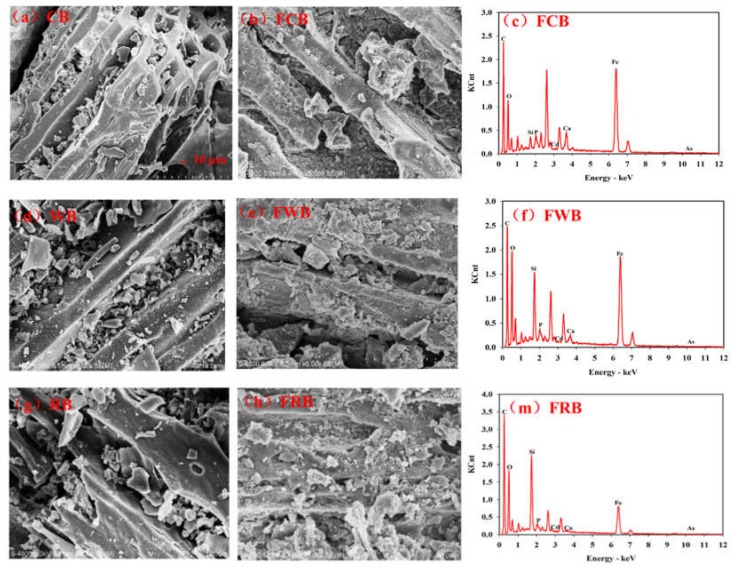
Scanning electron microscopy-EDS spectra of pristine (**a**: CB; **d**: WB; **g**: RB) and Fe-modified biochar (**b,c**: FCB; **e,f**: FWB; **h,m**: FRB).

The morphology and chemical composition of pristine and Fe-modified biochar materials were determined by SEM-EDS (Figure 1). As shown in the SEM images, biochar had porous and lamellar structure. Debris and impurities adhere to the surface of biochar. After modification, Fe-modified biochar had more roughness and granular or massive structures than pristine biochars. Significant peaks of Fe from Fe-modified biochar were shown in Figure 1c,f,m, and they were confirmed by the elementary composition analysis. As shown in Table S2, Fe contents increased from 0.1% to 34%, 28% and 12% for FCB, FWB and FRB, respectively. All biochar loaded lower contents of Cd(II) (≤1%) and As(V) (≤0.5%). Three pristine biochar had different values in pH and chemical elements. RB had the highest pH of 10.4, followed by WB (9.02) and CB (7.28). While Fe-modified biochar had lower pH values than pristine biochar. FCB had the highest pH of 6.99, followed by FWB (6.45) and FRB (6.23). CB and FCB both loaded highest fractions of element P, but lowest of element Si. According to the FITR spectra for the pristine and modified biochar (Figure S1), all types of biochar contained a number of carboxylic, carbonyl and hydroxyl groups (around 1620 cm^−1^, 1400–1000 cm^−1^), FeCl_3_ modification had little effect on the surface functional groups of biochar.

Biochar could sorb metals, such as Cd, Pb, and Cu etc., from both soil and water, mainly due to its physico-chemical properties [42]. SEM images of biochar with or without Fe modification showed that iron particles filled the empty micro or mesopores, thus reducing the porosity and surface area of biochar [43]. In addition, the types of feedstocks and pyrolysis temperatures also affected the surface area and porosity of biochar [8]. These physical properties contributed to the heavy metal sorption capacity of biochar, but not the most important. Chemical properties of biochar such as pH, surface charges and functional groups could mediate the metal sorption, mainly through the surface complexation, precipitation, electrostatic attraction, cation exchange and reduction mechanisms etc [44,45]. However, due to the limited metal sorption capacity of pristine biochar, more studies paid attention to the application of biochar modification [18]. Impregnation of iron salts with biochar was the conventional method for enhancing the heavy metal (Cd, Pb, As etc.) sorption capacity. Fe-impregnation led to the hydrolysis of FeCl_3_ and the production of HCl, which caused lower pH values of biochar after modification [43]. As reported, Fe-modified biochar had lower negative charges, thus resulting in the higher electrostatic sorption of anion contaminants such as arsenate (As(V)) [24]. The formation of Fe-O-As(V) complexes were also responsible for the sorption of As(V) onto the Fe-modified biochar [46]. In comparison with the other two biochar, FRB loaded a lowest amount of Fe. Reasons possibly due to the physicochemical properties of RB and experimental process. The reduction in pH values of modified biochar was controlled by the surface functional groups, and the CEC (cation exchange capacity) values of biochar [47]. The loss of surface inorganic C during Fe-impregnation could destroy the porosity of biochar surface [43]. The surface oxygen-containing functional groups, such as -COOH and -OH, could sorb the heavy metal contaminants, mainly through the surface complexation and cation exchange mechanisms [48,49]. However, no significant changes in these functional groups were found in the surface of biochar before and after Fe modification. 

### 3.2. Effect of Biochar on Metal Immobilization

**Figure 2:**
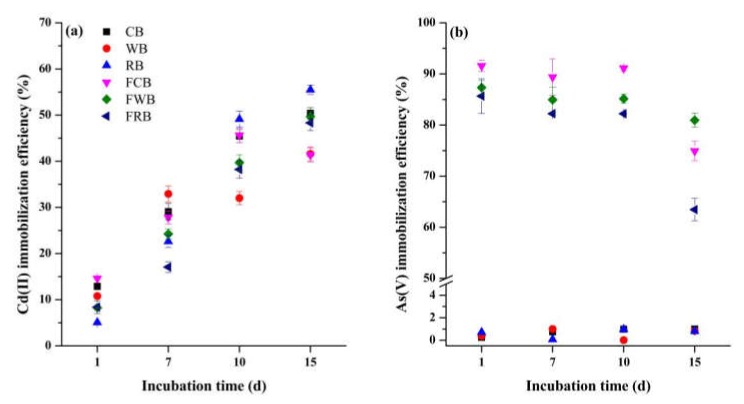
Effects of remediation time on soil Cd(II) and arsenate As(V) immobilization. (**a**) Cd(II) immobilization efficiency of various biochar and (**b**) As(V) immobilization efficiency of various biochar.

Cd(II) immobilization efficiency of 5% (w/w) biochar varied with types and incubation time (Figure 2a). In general, biochar increased the soil Cd(II) immobilization efficiency with incubation time. At the first day of incubation, Cd(II) immobilization efficiency of CB, WB and RB was 12.89, 10.78 and 5.08%, respectively. These efficiencies increased to 50.38, 41.59 and 55.49% at 15 days of the incubation. Similar phenomenon was observed for treatments of Fe-modified biochar. During 7–15 days of the incubation, Cd(II) immobilization efficiencies of FCB and FRB were always lower than their pristine biochar. While FWB amendment caused higher Cd(II) immobilization efficiency of 49.68% than WB amendment (41.59%). Among biochar, RB amendment had the highest Cd(II) immobilization efficiency of 49.12%, 55.49% at 10 and 15 day of the incubation, respectively. Different from Cd(II), As(V) immobilization efficiency of pristine biochar were lower than 1%, and slightly varied with incubation time. As(V) immobilization efficiencies of Fe-modified biochar were larger than 82% during 1–10 day of the incubation. FCB amendment caused the highest efficiency of As(V) immobilization (> 89%), followed by FWB and FRB. Sharp declines in the efficiencies were observed at 15 day of the incubation. FWB amendment caused a highest efficiency of 80.94%, followed by FCB (74.93%) and FRB (63.46%). Results showed that the optimal remediation time was 10 d for Cd-As contaminated soil.

**Figure 3:**
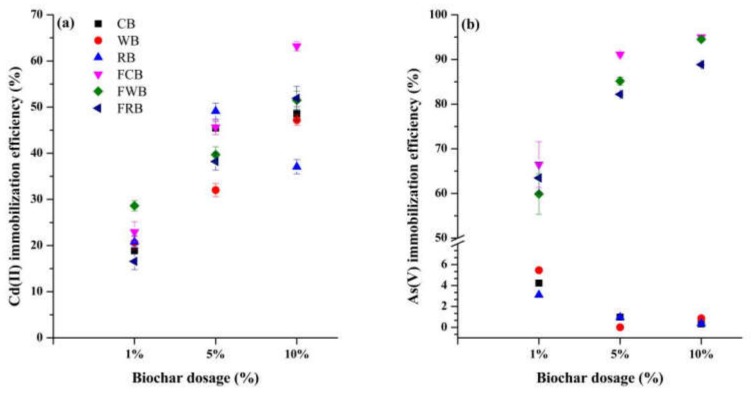
Effects of changes in amendment levels on soil Cd(II) and As(V) immobilization. (**a**) Cd(II) immobilization efficiency of various biochar and (**b**) As(V) immobilization efficiency of various biochar.

Effects of biochar dosage on soil Cd(II) and As(V) immobilization were studied after 10 days of incubation (Figure 3). Cd(II) immobilization efficiencies of biochar increased with dosage from 1% to 10% (Figure 3a). At a dosage of 1%, efficiencies of CB, WB and RB were 18.86, 20.62, and 20.81%, respectively. These efficiencies increased to 48.57, 47.20, and 30.05% at a dosage of 10%, respectively. FCB and FWB amendments always caused higher efficiency than CB and WB, respectively. FCB amendment had the largest efficiency of 63.21% at a dosage of 10%. FRB amendment had a higher efficiency (51.91%) than RB (37.05%) that only observed at a dosage of 10%. Different from Cd(II), pristine biochar caused relatively lower As(V) immobilization efficiency (<6%) than Fe-modified biochar (Figure 3b).. These efficiencies decreased to zero near at the dosage of 10% for pristine biochar. However, As(V) immobilization efficiency of Fe-modified biochar increased with dosages. FCB caused the highest efficiency of 66.47% at a dosage of 1%, followed by FRB (63.48%) and FWB (59.88%). While at the dosage of 10%, FCB, FWB and FRB had an efficiency of 95.10, 94.51 and 88.84%, respectively. Results showed that the optimal dosage is 10%, especially for Cd-As contaminated soil.

TLCP method was reported to be a suitable method for assessing heavy metal bioavailability, which can be reflect the heavy metals immobilization efficiency of amendments [38]. The dynamic results of metal immobilization efficiency in Cd-As contaminated soils indicated that behaviors of sorption competition, surface complexation and precipitation between metals and active sites are complex dynamic processes. The negatively charged biochar could enrich Cd(II) and repel As(V). Differences in the O-containing functional groups, surface charge compositions among three biochar contributed to their capacities in sorption of metals [8]. Different from single metal contamination, sorption antagonism and synergy of As(V) and Cd(II) onto biochar amended soils could be more complex [24,50]. Study of Zhang et al. [24] showed that coexistence of As(V) in wastewater increases the removal of Cd(II) (>50%) by Fe-modified biochar. The negatively charged sites and formation of Fe-As-Cd complexation both contributed to the removal of Cd(II) [24]. Similar results were observed in the study of Liang et al. [51], who found that the presence of As(V) increases the Cd(II) adsorption by variable charge soils, mainly through the electrostatic effect. Though the detection of SEM-EDS, Beesley and Marmiroli [42] proposed that arsenic sorption may only occur on the outer surfaces of biochar. Cd(II) sorption onto biochar surface could occurred at both outer and inner surfaces, thus specific sorption rather than the ion exchange behavior was responsible for Cd(II) immobilization [42,50]. Biochar was reported to have a dual role in the sorption of aluminum, the co-precipitation of Al with silicate particles (KAlSi_3_O_8_) could cause charge reversal of biochar surface [52]. We speculated a similar phenomenon that surface charge reversal of Fe-modified biochar would happen after the inner sorption of Cd(II). Therefore, the surface sorbed As(V) decreased with the sorption of Cd(II) onto Fe-modified biochar. According to our results, Fe amounts loaded on biochar surfaces modulated the immobilization of Cd(II) and As(V). The highest rate of 10% biochar amendment provided more active sites and surface charges for metal immobilization. As shown in Table S2, FCB loaded the highest percentage of 34% Fe and highest pH of 6.99 when compared with FWB and FRB. FCB was chosen as the optimal amendment for Cd-As contaminated soil. 

### 3.3. Metal Species and Soil Properties Respond to Biochar Amendments

**Figure 4:**
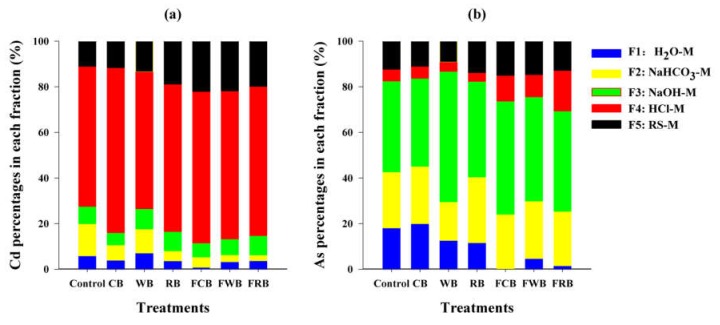
Changes of soil Cd and As fractions in soil before and after remediation. (**a**) Cd percentages in each fraction and (**b**) As percentages in each fraction.

Total heavy metal contents were limited in predicting their bioavailability and ecotoxicity. Studies proved that chemical forms distribution of metals into soil (minerals, particles and DOM etc.) determine their bioavailability [1,53]. Wan et al. [54] suggested that the Shiowatana sequential extraction procedure (SEP) is more suitable for arsenic fractionation and predicting its bioavailability and ecotoxicity. Therefore, in our study metal fractions were extracted according to the Shiowatana SEP. Metal fractions were sequentially extracted from Cd-As contaminated soils, which were amended with a dosage of 10% pristine or Fe-modified biochar for 10 days, respectively (Figure 4). Cd(II) presented in water-soluble (Cd_F1_) and surface absorbed (Cd_F2_) fractions accounted for 20.0% in control soil (Figure 4a). These two fractions were proven to be the most labile fractions and can be easily transferred into plants [54,55]. Biochar amendment significantly decreased their proportions, thus causing Cd(II) immobilization and reducing its bioavailability in contaminated soil. For example, RB, among pristine biochar, amendment caused a lowest percentage of 8.03% in Cd_F1+F2_. FCB amendment caused the lowest percentage of 5.34% in Cd_F1+F2_ among six biochar. Most of Cd(II) presented in OM and sulfides (Cd_F4_ > 60%), followed by the residual (Cd_F5_ > 10%). When compared with the pristine biochar amendment and control soils, Fe-modified biochar amendment increased the percentages of Cd(II) presented in Fe-Al (oxides and hydroxides) (Cd_F3_), Cd_F4_ and Cd_F5_ fractions. After 10 days of incubation, FCB amendment caused a highest percentage of 88.52% in Cd_F4+F5_, followed by FWB (86.75%) and FRB (85.24%). Different from Cd, most of As presented in Fe-Al (oxides and hydroxides) (As_F3_ > 35%), followed by the surface absorbed (As_F2_ > 15%) fraction (Figure 4b). Fe-modified biochar amendment caused lower percentages in As_F1+F2_ (24.13–29.92%), than pristine biochar amendment (29.58–45.17%) and control (42.70%) soils. However, FCB amendment caused a highest percentage of 75.87% in As_F3+F4+F5_, followed by FRB (74.57%) and FWB (70.08%). 

As shown in Table 1, soil pH values after Fe-modified biochar amendment were nearly 8% higher than pristine biochar amendment and control soils. Soil inorganic nitrogen contents after Fe-modified biochar amendment decreased by 57.3% (FRB), followed by RB amendment (28.77%). These higher soil pH and less negatively charged surfaces of Fe-modified biochar, contributing to the loss of soil ammonium nitrogen content. Different from soil inorganic N, the dissolution of P from biochar and formation of Cd_3_(PO_4_)_2_ co-precipitation both happened during biochar amendment [56,57]. Not only Cd, but also phosphorus anions are bound to many positively charged particles such as calcium (Ca) and iron hydroxides [58]. When compared with CaP, FeP was reported to be less available phosphates [59]. The formation of FeP in agricultural soil could reduce the available phosphorus to plants and soil microorganisms, thus increasing the agricultural production cost [59]. In comparison with control, biochar amendment reduced the contents of soil available P, except for the FCB treatment. These soil properties were changed with biochar amendment, also directly or indirectly affected the metal immobilization. Soil DTPA extractable Fe (DTPA-Fe) contents were significantly increased by maximally 1.68 folds with the addition of Fe-modified biochar (FCB). Pristine biochar amendment all decreased the contents of soil DTPA-Fe. A similar phenomenon was reported by Su et al. [38]. The reason was possibly due to the sorption of available Fe onto biochar. These increased contents of soil DTPA-Fe could facilitate the distribution of metal into Fe oxides and hydroxides. Fe is an important element for plant growth and development [60]. Usage of Fe-modified biochar in the immobilization of soil heavy metals, not only decreasing metal bioavailability but also improving the soil quality for revegetation and restoration. Further study is still needed to investigate the responses of soil organisms and microbial community to the remediation of Fe-modified biochar. Then we can evaluate the application of Fe-modified biochar in remediation of soil Cd-As contamination more properly. 

## 4. Conclusions

Fe-modified biochar was applied to immobilize soil Cd(II) and As(V), in comparison with the pristine biochar. Among three types of biochar, FCB at 10% was the most effective in enhancing both Cd(II) and As(V) immobilization efficiency after 10 days of the incubation. Biochar amendment decreased the available metal of water-soluble (F1) and surface absorbed (F2) fractions. Most Cd presented in OM and sulfides (F4) fraction after FCB amendment, while most As presented in Fe-Al (oxides and hydroxides) (F3) fraction. FCB amendment had higher soil pH values and DTPA-Fe contents than pristine biochar. No significant increase of soil available nutrient was noticed after FCB amendment. FCB, combining the advantages of biochar and Fe^3+^, is feasible to the remediation of Cd-As contaminated soil. Therefore, application of FCB enabled the reuse of organic waste, contaminant immobilization, and soil quality improvement. However, the immobilization efficiency and application safety of FCB still needed to be confirmed with the responses of organisms under long-term field experiments.

## Figures and Tables

**Table 1:** Soil properties after optimized amendment.

Treatment	pH	Inorganic *N*	Available *P*	DTPA-Fe
		mg/kg		
Control	4.86 ± 0.001a	112 ± 0.91g	9.19 ± 0.11d	116 ± 1.81c
CB	4.89 ± 0.01a	90.2 ± 1.38f	8.18 ± 0.05c	81.6 ± 2.64b
WB	4.82 ± 0.08a	82.4 ± 0.16e	7.11 ± 0.47b	71.7 ± 1.53a
RB	4.85 ± 0.04a	80.0 ± 0.21d	6.44 ± 0.47a	82.3 ± 4.21b
FCB	5.25 ± 0.02c	67.5 ± 0.04c	9.25 ± 0.35d	195 ± 4.11f
FWB	5.20 ± 0.01b	58.9 ± 0.37b	6.77 ± 0.47a	181 ± 3.29e
FRB	5.21 ± 0.04bc	48.0 ± 0.45a	7.02 ± 0.59b	176 ± 2.69d

Different lowercase among treatments denoted the significant difference at *p* < 0.05 (LSD).

## Supplementary Materials

The following are available online at https://www.mdpi.com/1660-4601/17/3/827/s1, Figure S1: FTIR spectral data for pristine biochars (CB, RB, WB) and Fe-modified biochars (FCB, FRB, FWB), Table S1: Physicochemical properties of the Cd-As contaminated soil, Table S2: pH and element composition (wt, %) of three biochar with or without FeCl_3_ modification.
