# Supplementary material for: Simultaneous Immobilization of Soil Cd(II) and As(V) by Fe-Modified Biochar
Source: Int J Environ Res Public Health. 2020 Jan 28;17(3):827. doi: 10.3390/ijerph17030827 (PMC7037325; doi:10.3390/ijerph17030827)
Supplement: Supplementary file 1 [file ijerph-17-00827-s001.pdf]

International Journal of
Environmental Research and Public Health

Article

Simultaneous Immobilization of Soil Cd(II) and As(V) by Fe-Modified Biochar

Yi-min Wang [1], Shao-wei Wang [1], Cheng-qian Wang [1], Zhi-yuan Zhang [1], Jia-qi Zhang [1], Meng Meng [1], Ming Li [2,*], Minori Uchimiya [3] and Xu-yin Yuan [1,*]

1 Key Laboratory of Integrated Regulation and Resource Development on Shallow Lakes, Ministry of Education, College of Environment, Hohai University, Nanjing 210098, China; wangym@hhu.edu.cn (Y.W.); w1205788761@126.com (S.W.); wsygsldxxn@163.com (C.W.); zaysjzzyyj@163.com (Z.Z.); zzjjqqhhu@163.com (J.Z.); mmxxff1224@163.com (M.M.)
2 Huatian Nanjing Engineering & Technology Corporation, Nanjing 210019, China
3 USDA-ARS Southern Regional Research Center, 1100 Robert E. Lee Boulevard, New Orleans, LA 70124, USA; sophie.uchimiya@usda.gov
* Correspondence: ming880609@126.com (M.L.); yxy_hjy@hhu.edu.cn (X.Y.)

Received: 3 January 2020; Accepted: 26 January 2020; Published: date

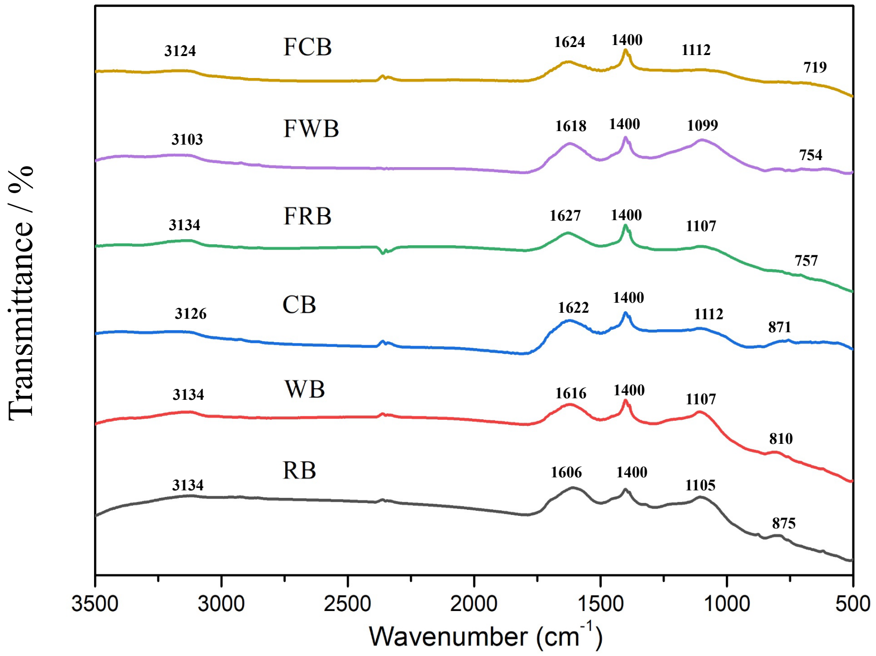

Figure S1. FTIR spectral data for pristine biochars (CB, RB, WB) and Fe modified biochars (FCB, FRB, FWB).

Table S1. Physicochemical properties of the Cd-As contaminated soil.

	pH	OM	TN	TP	Cd	As	Total Fe	Soil particle composition (%)		
			g/kg			mg/kg	g/kg	2-0.05mm	0.05–0.002mm	<0.002 mm
Xianning soil, Hubei	4.86	29.5	1.81	0.39	2.15	243	22.1	9.86	65.7	24.4

Table S2. pH and element composition (wt, %) of three biochar with or without $FeCl_3$ modification.

	CB	WB	RB	FCB	FWB	FRB
pH	7.82	9.02	10.4	6.99	6.23	6.45
C	76.21	68.34	58.5	43.14	40.9	52.39
O	19.83	20.77	31.66	17.86	24.47	27.3
P	1.63	0.50	0.88	1.25	0.97	0.78
Si	0.41	8.68	7.55	0.73	4.31	6.17
Ca	0.78	0.63	0.55	2.09	0.65	0.19
Mg	0.37	0.45	0.17	0.00	0.06	0.02
Fe	0.13	0.10	0.12	34.24	28.1	12.32
Cd	0.49	0.43	0.40	0.20	0.65	0.28
As	0.13	0.10	0.18	0.49	0.30	0
